# Measles Elimination: Identifying Susceptible Sub-Populations to Tailor Immunization Strategies

**DOI:** 10.3390/v11080765

**Published:** 2019-08-20

**Authors:** Peter Kreidl, David Ammerer, Reinhard Würzner, Anita Luckner Hornischer, Dorothee von Laer, Wegene Borena

**Affiliations:** 1 Department of Hygiene, Medical Microbiology and Public Health, Medical University Innsbruck, 6020 Innsbruck, Tyrol, Austria; 2 Department of Pneumology, Regional Hospital Hochzirl-Natters, 6161 Natters, Tirol, Austria; 3 Division of Virology, Medical University Innsbruck, 6020 Innsbruck, Tyrol, Austria; 4 Regional Public Health Authority, 6020 Innsbruck, Tyrol, Austria

**Keywords:** measles, serosurvey, vaccine coverage, surveillance

## Abstract

Measles elimination has been identified as a public health priority in Europe for a long time but has not yet been achieved. The World Health Organization (WHO) recommends identification of susceptible sub-populations to target supplementary immunization activities. We used three different sources of information: retrospective samples investigated for measles IgG between 1997 and 2016, vaccine coverage data from the existing electronic registry for birth cohorts 2015 to 1999, and surveillance data from 2009 until 20 July 2019. We calculated susceptibility by birth cohort using seroprevalence data, adjusting vaccine coverage data with reported effectiveness (93% for the first and 97% for the second dose, respectively), and compared it with measles incidence data, aggregated by birth cohorts and districts. Susceptibility levels for persons 10–41 years (birth cohorts 2007–1976) were 10.4% and thus far above the recommended values of WHO (5%). Older birth cohorts were sufficiently protected. Districts with the highest susceptibility estimates corresponded with districts with the highest incidence rates. Birth cohorts with susceptibility levels > 10% showed a 4.7 increased relative risk of having had more than one measles case. We conclude that retrospective serosurveys are a cheap and useful approach in identifying susceptible sub-populations, especially for older birth cohorts whose coverage data remain scarce.

## 1. Introduction

Measles elimination in the WHO Region for Europe was already planned for 2010 but has not yet been achieved [1,2]. The number of reported measles cases in the region increased 15 times between 2016 (the year with the lowest number of cases recorded) and 2018 [3]. Several issues have contributed to this phenomenon, one of them being vaccine hesitancy- a factor identified as one of the 10 key health threats in 2019 [4]. WHO recommends to diagnose the determinants of insufficient vaccine uptake and to tailor vaccination strategies [5]. Susceptibility levels below 5% in children 10 years and older, below 10% in children age five to nine years, and below 15% in children 24 months to 4 years are needed to eliminate measles [6].

In Austria, mandatory reporting of suspected measles cases has been implemented in 2006 [7]. The first measles containing vaccines (MCV) were licensed in 1963 in the US [8] and, since 2001, more than 2 billion doses were administered globally [9]. Bivalent (with mumps) MCV was recommended in 1974 in Austria, and the use of two doses of trivalent (measles–mumps–rubella) vaccine (MMR) was recommended in 1994 [10]. Since 1985, MCV was administered free of charge for children up to 15 years of age and since 2015 for all age groups [11].

Vaccine coverage is estimated by the administrative method in Austria. Since 2016, an agent-based simulation model has been implemented to better assess the number of susceptible persons to measles [12].

In Tyrol, one of Austria’s nine regions with 739,139 inhabitants in 2016 [13], the estimated coverage of the birth cohorts 2000 to 2009 was 88.2% with one dose and 76.5% with two doses of measles containing vaccine in 2012 [14]. Since 2000, an electronic vaccination registry has been fully implemented in Tyrol.

As of 2014, the Tyrolean government has required proof of two doses of MCV from all health care workers (HCW) in Tyrolean hospitals. Those who lack documented evidence of two doses of MMR are required to undergo serological testing. The same applied to all medical students.

The WHO provides guidance to seroprevalence surveys to better identify pockets of susceptible populations, and recommends their use if surveillance was only recently implemented and when coverage data are less reliable for older age groups [15]. Nevertheless, serosurveys as recommended by WHO are costly, logistically difficult to conduct, and require substantial time commitment as well as ethical approval. The objective of our work was to assess the usefulness of retrospective seroprevalence data and to describe the proportion of measles susceptible persons by birth cohort and district to better understand sub-populations that are most at risk. This knowledge may help to tailor awareness raising activities as mentioned in the national measles elimination plan [16].

## 2. Materials and Methods

In order to identify sub-populations that are most at risk for measles, we assessed the population immunity using three different sources of information: The results of an analysis of the MCV coverage data extracted from the regional electronic vaccination registry, the results from an ad-hoc retrospective serosurvey conducted in 2017 and the most recent surveillance data from the electronic reporting system EMS [17].

### 2.1. Retrospective Seroprevalence Survey

We used retrospective data of all persons who were investigated for measles IgG antibodies between 1997 and 2016 in the Division of Virology at the Medical University of Innsbruck (Sheet S1). This laboratory received the majority of blood samples for measles serology testing in Tyrol which were approximately 1300 samples per year. Testing was usually requested for unspecific exanthema with or without fever. The information available at the laboratory did neither include detailed clinical information nor information about the vaccination status. IgM results were not considered in our analysis because detection of recent infection was not our primary objective. Samples were classified as positive/negative according to cut-off values described by the respective manufacturer. Borderline values were excluded. Between 1 January 1997 and 19 November 2016 an enzyme immunoassay (ELISA; Enzygnost^®^ Anti-Measles Virus/IgG, Siemens, Marburg, Germany) and since then a chemoluminent immunoassay (CLIA, LIAISON^®^ Measles IgG, Diasorin, Saluggia, (VC), Italy) were used. A sufficient number of parallel tests were conducted using both kits during the transition phase from ELISA to CLIA and results were comparable between the two kits. The last available laboratory result of each individual obtained between 1 January 1997 and 31 December 2016 was included. The exclusion criteria included the following: (a) place of residency outside Tyrol or not available, (b) date of birth before 1917 or not available, (c) inconclusive laboratory result, or (d) potential immunodeficiency. Potential immunodeficiency was defined as admittance to an intensive care unit (ICU) department or any other department, where patients were likely to be immunocompromised at the time of the sampling, such as oncology, transplant, or hematological wards. Furthermore, we excluded all HCWs and medical students tested for measles IgG from our analysis. Both groups who could not provide documented evidence of vaccination with two doses of MCV were requested by the regional authorities to confirm immunity by IgG testing.

The age of the participants was calculated as of 31 December 2016. Susceptibility to measles was assumed to be unchanged since the time of the last investigation.

We defined birth cohorts with susceptibility levels higher than 10% as high risk cohorts.

Seroprevalence data were extracted from the virological laboratory software in an anonymized way and analyzed using Epiinfo^TM^ (Version 7.2.2.2, CDC Atlanta, GA, USA). We calculated the susceptibility by birth cohort and district, using the mid population data between 1996 and 2016 [13].

The study was approved by the ethical committee of the Medical University of Innsbruck under EK Nr: 1082/2017.

### 2.2. Susceptibility Estimates from the Electronic Vaccination Registry

We extracted the MCV coverage data from the electronic vaccination registry of Tyrol in July 2018 for all available birth cohorts (1997–2013) in an aggregated format (Sheet S3). Data from 1999 were incomplete but were still included in the analysis. MCVs were trivalent MMR (measles–mumps–rubella) vaccines. We used the denominator data available from Statistik Austria from 2016 [13] to calculate coverage. We calculated the susceptibility levels using MCV coverage and assumed a vaccine effectiveness of 93% for the first and 97% for the second dose. Susceptible persons were stratified by number of MCV doses received and district of residency.

### 2.3. Reported Measles Cases in Tyrol

We used the case-based measles surveillance reports between 2003 and 2019 from Tyrol (Sheet S2) and compared them with the susceptibility levels by birth cohort obtained from the seroprevalence survey. We used the number of reported measles cases between 2009 and 2019 (prior to 2009 the district of cases was not available) to calculate the mean annual incidence for the 10.6-year period by district (number of cases per 1 million inhabitants). We compared the district specific incidence with the susceptibility estimates from the serosurvey. We defined birth cohorts with more than one measles case between 2009 and 2019 as high incidence birth cohorts. We calculated the relative risk and the 95% confidence interval for birth cohorts with susceptibility levels > 10% (exposure) with more than one reported measles case during the observation period (outcome: high incidence birth cohorts). 

## 3. Results

### 3.1. Retrospective Seroprevalence Survey

**Figure 1:**
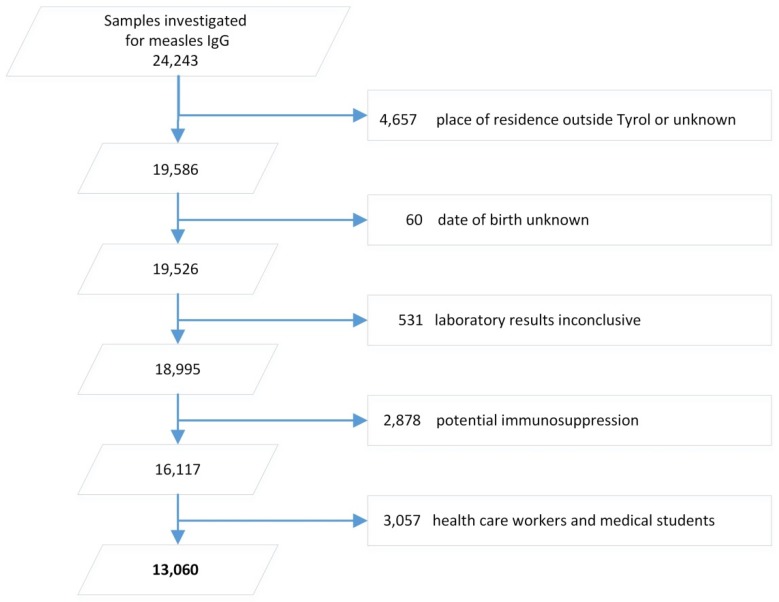
Sample size of investigated patient samples in Tyrol, 1997–2016 and final sample size for analysis after application of inclusion and exclusion criteria.

Between 1 January 1997 and 31 December 2016, 24,243 samples were investigated for measles IgG. After application of the exclusion criteria, 13,060 individual records remained and were included in our analysis (Figure 1).

**Figure 2:**
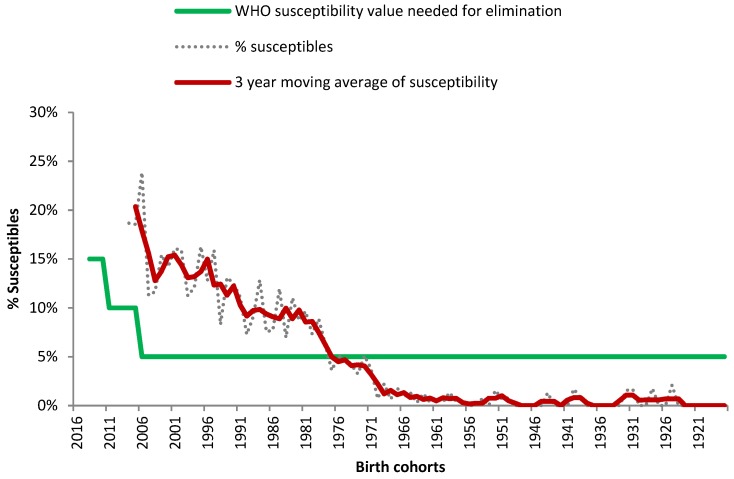
Proportion of susceptible persons by birth cohort, 3-year moving averages and WHO recommended susceptibility values needed for elimination (birth cohorts 1917–2016).

Women accounted for 54% of participants (*n* = 7,057); for 1.5% (*n* = 195) gender information was not available. The highest number of participants was in the age group 40 to 49 years (as of 31 December 2016) (Table 1). Children between 24 months of age and 4 years were underrepresented (Figure 2).

All nine districts of Tyrol sent samples to the laboratory. The mean proportion of the represented population by district was 1.7% ranging from 0.8% to 2.6%.

Among all participants 5.9% were susceptible to measles. Susceptibility levels were age dependent (Table 2). The highest susceptibility levels were, as expected, in the most recent birth cohorts. The age dependent WHO recommended susceptibility levels needed for elimination (5–15%) [6] were achieved by birth cohorts 1978, 1979, and 1976 and older (Figure 2). Susceptibility levels for persons 10–41 years (birth cohorts 2007–1976) were 10.4% and therefore far above the values recommended by WHO (5%).

Thirty-seven of 99 birth cohorts (excluding the birth 2016 cohort due to non-eligibility for vaccination) did not achieve the necessary immunity levels needed for elimination. Among them, the mean difference between expected WHO susceptibility requirements and observed susceptibility levels was 17.6% (median 12.9%; range: 5.3–75.0%). The five most recent birth cohorts (2011–2015) showed levels of 25% or higher above the expected values. Ten birth cohorts (1977,1980–1982, 1985, 1987–1988, 1990–1991, and 1995) showed susceptibility levels between 5–9%, and 22 birth cohorts (1983, 1984, 1986, 1989, 1992–1994, 1996–2010) between 1% to 4% deviating from the target, respectively. 

Less females were susceptible to measles (5.1%) compared to males (7.1%) (*p* < 0.0001). The mean proportion of susceptible persons per district varied between 5.0% and 8.6%, with a mean of 6.4% and a median of 6.0%.

### 3.2. Coverage Data with Two Doses of Measles Containing Vaccine from the Tyrolean Electronic Vaccine Registry

**Figure 3:**
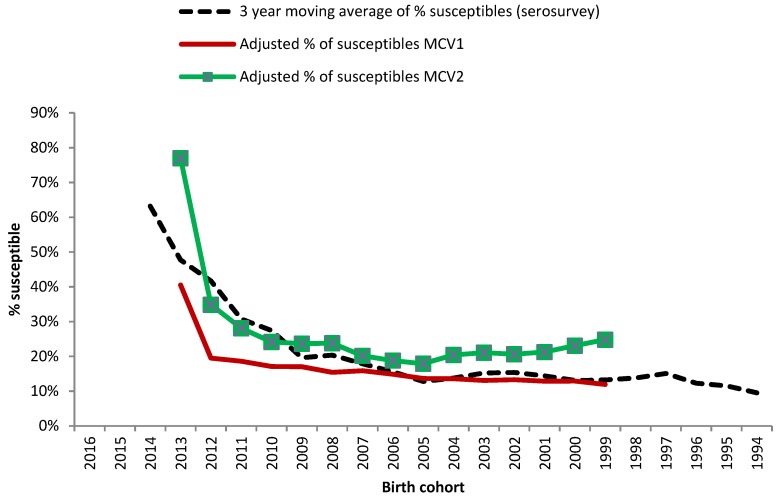
Proportion of susceptible persons by birth cohort (1994–2014): comparison of 3-year moving average susceptibility levels of seroprevalence data, adjusted vaccine coverage data for one or two doses of a measles containing vaccine (MCV), assuming a vaccine effectiveness of 93% for the first and 97% for the second dose.

The adjusted susceptibility levels by birth cohort using one (MCV1) or two doses (MCV2) of MCV both followed the pattern of the 3-year moving average seroprevalence susceptibility data, especially for MCV1 for birth cohorts 2009 and older. The mean difference between seroprevalence data and first dose adjusted susceptibility was 5% (median 2%, range −1% to 22%), and the mean for the second dose was −5% (median −5%; range, −29% to 7%), respectively. While the estimated proportion of susceptible population with one dose of vaccine was below the data from the seroprevalence survey the estimated susceptibility with two doses of MCV was above the seroprevalence estimates—except for the birth cohorts 2010 to 2012 (Figure 3).

### 3.3. Reported Cases of Measles in Tyrol

Between 2003 and 2019, a total of 99 measles cases were reported. Since 2008, peaks with a maximum of 16 cases in 2011 were observed every 3–4 years (Table 3). Detailed case information was only available for cases since 2009.

Among the 77 cases reported since 2009, the mean age at the time of diagnosis was 20.5 years (median 21 years; range 7 months to 48 years). Females accounted for 44% (*n* = 34) of cases.

The mean annual number of cases for 2009–2019 per district varied between 0 and 19 cases, the mean annual incidence between 0 and 20.3 cases per 1 million inhabitants (mean 9.7, median 7.5). The highest incidence was reported from the district with the highest proportion of susceptible population from the seroprevalence data (Table 4)

Among the 100-year birth cohort band, 72 birth cohorts had susceptibility levels below 10% and 61 birth cohorts below 5%. The relative risk of having observed more than one measles case per district between 2009 and 20 July 2019 in districts with susceptibility levels > 10% was 4.7 (95% CI: 2.52–8.52).

## 4. Discussion

We assessed the susceptibility levels for measles among the Tyrolean population using three sources of information: a retrospective analysis of already available serological data for measles IgG (serosurvey), vaccine coverage data from the electronic vaccination registry and surveillance data from the mandatory electronic reporting system.

We found that the WHO recommended susceptibility thresholds of maximum 15% in children 24 months–4 years, 10% in persons 5–9 years, and 5% in persons >10 years were not achieved. Susceptibility was age dependent and the threshold of less than 5% was only reached by birth cohorts 1976 (41 years of age as of 31 December 2016), 1978, and 1979 or older. Susceptibility levels for persons 10–41 years (birth cohorts 2007–1976) were twice as high as the recommended values of WHO of 5% for these age groups.

More than one-third of the population was considered to be immune with susceptibility levels below the requested age-dependent thresholds. The highest susceptibility levels were identified among the most recent birth cohorts until birth cohort 2011, with susceptibility levels differing between 15–50% from the WHO target. Ten birth cohorts revealed susceptibility levels between 5% and 9% and further 22 birth cohorts levels between 1% and 4% above the expected WHO targets. Many member states of the European Union did not achieve the requested WHO goals and susceptible persons accumulated over time [18]. Even a slight increase in MCV uptake was associated with significant reduction of overall burden of disease [19].

Significantly more males were susceptible to measles compared to females. This uneven gender distribution was also confirmed by surveillance data. Such inequities are not yet addressed through vaccination policies [20] and studies suggest there are no major sex differences in IgG production following vaccination [21].

We identified several limitations of our seroprevalence study: due to the very long observation period, the likelihood of losing detectable antibody levels—especially of persons who were vaccinated with only one dose of MCV—has to be considered and might have biased our results. This would probably not apply for persons who had either experienced natural infection, received two doses of MCV or were re-exposed. This may have been of less influence, as participants from nearly all birth cohorts were investigated recently. In fact, the long time span of the serosurvey resulted in a large sample size and thus allowed more precise estimates. Estimates also became more reliable due to careful exclusion of ambiguous data and they were based on well validated test kits in an accredited laboratory. Despite the fact the only few seroprevalence data of recent birth cohorts were available, the estimation of susceptibles by the two different approaches, retrospective seroprevalence data, and estimation of susceptibles using coverage data corresponded quite well. As expected, coverage with one dose of MCV was constantly higher compared to coverage with two doses and thus susceptibility was inversely correlated. The long observation period may have also resulted in an underestimation of susceptibility in more recent birth cohorts due to very small numbers. Using coverage data from the electronic registry has resulted in a much better estimate of susceptibles of more recent birth cohorts. For the assessment of susceptibility with one or two doses of MCV we used the reported vaccine effectiveness for each dose as reported by the Centre for Diseases Control (CDC), Atlanta [22]. The assumption of 93% vaccine effectiveness for the first dose and 97% of the second dose was very conservative as higher vaccine effectiveness was reported by other authors [23,24].

Another limitation of our seroprevalence data was that despite the entire region being covered, districts that are physically closer located to the laboratory were overrepresented and districts further away sent fewer samples. This is important as Tyrol is known to be a region with a high prevalence of vaccine hesitant persons [25]. This may have resulted in a greater inaccuracy from districts further away from the capital. Nevertheless, the highest incidence districts calculated from surveillance data were in line with the highest susceptibility levels by district from the seroprevalence data. As vaccine coverage data were only available in aggregated format, we could not compare incidence with coverage by district.

The differences of susceptibility by birth cohort with seroprevalence and estimated adjusted susceptibility data from the electronic vaccination registry corresponded well in their pattern, and the mean varied between −5% for the second dose and 5% with the first dose. Susceptibility levels of seroprevalence data were higher in recent birth cohorts until 2009 compared to susceptibility calculated using coverage data. The latter was probably due to the small sample sizes of recent birth cohorts in the seroprevalence survey.

The estimates of susceptible persons from the agent-based simulation model at a national level identified that approximately 70% of 19–30 year-old persons were protected with two doses of MCV as of 2018. In our data the increase of susceptibility using the second dose adjusted susceptibility started already from the birth cohort 2005 until birth cohort 1998 [12]. This may be due to regional differences.

Limitations of our coverage data were the different sources of numerator and denominator data. Participants who moved out of the region or died were still included in the numerator but were excluded from the denominator, in which only resident persons were reported. This might have led to an overestimation of vaccine coverage.

Limitations of our surveillance data were that detailed information was only available since 2009. As older age groups were more affected in recent years in many countries, this may have resulted in underestimation of attack rates of younger individuals. If a virus is introduced to a susceptible population, cluster cases are known to occur in similar age groups. Therefore, the short observation period of our surveillance data may have reflected this phenomenon. Nevertheless, birth cohorts with more than one case reported were significantly positively associated with susceptibility levels > 10%.

Targeted catch-up campaigns of the identified birth cohorts focusing on high incidence districts would definitely be crucial to close immunization gaps and reduce the number of susceptibles but raising awareness and social mobilization of the target groups were shown to be difficult. In our experience, when mandatory vaccination has not been implemented and no incentives for vaccination were provided, participation in catch-up campaigns remained unsatisfactory [25].

We conclude the Tyrolean population is not sufficiently protected against measles and additional measures such as targeted supplementary immunization activities (e.g., targeted catch-up campaigns) are necessary to achieve elimination. This was also underlined by the fact that the majority of recently reported measles cases corresponded well with the birth cohorts with highest susceptibility levels. Priorities should be given to districts and birth cohorts with the lowest susceptibility, considering the WHO recommended values.

Our seroprevalence data suggested that additional immunization activities should target birth cohorts between 2011 and 2015 as a first priority and birth cohorts between 1998 and 2010 as a second priority.

Furthermore, our results suggest that birth cohorts before 1976 are not target groups for supplementary immunization activities as the majority of them are probably immune due to natural infection. In 2019, we conducted several activities mainly during the European Immunization Week to increase awareness and enhance uptake targeting the identified birth cohorts and districts, such as social mobilization and information campaigns in schools and universities, including health care workers.

We conclude that using different sources of information such as vaccine coverage data, surveillance data, and retrospective seroprevalence data is useful to identify pockets of susceptibles and retrospective seroprevalence data could be an additional cheap and useful source of information to better identify sub-populations, such as birth cohorts and geographical areas with increased measles susceptibility. This will especially apply for older birth cohorts, whose coverage data are lacking.

## Figures and Tables

**Table 1:** Characteristics of study participants: age (calculated as of 31 December 2016), birth cohort, and proportion of participants by birth cohort.

Birth Cohorts	Age Groups	N Participants	%
2016–2013	24 months to 4 years	45	0.3
2012–2008	5 to 9 years	331	2.5
2007–2003	10 to 14 years	733	5.6
2002–1993	15 to 24 years	1780	13.6
1992–1978	25 to 39 years	2701	20.7
1997–1958	40 to 59 years	4186	32.1
1957–1917	60+ years	3284	25.2
	TOTAL	13,060	100

**Table 2:** Measles susceptibility levels by birth cohort and age group and WHO recommended maximum susceptibility values necessary for elimination.

Birth Cohorts	Age Groups	% Susceptible	Maximum WHO Recommended Value
2014–2013	24 months to 4 years	60.0	15
2012–2008	5 to 9 years	23.9	10
2007–2003	10 to 14 years	14.9	5
2002–1993	15 to 24 years	13.0	5
1992–1978	25 to 39 years	8.8	5
1997–1958	40 to 59 years	1.9	5
1957–1917	60+ years	0.4	5
Total		5.9	

**Table 3:** Number of reported measles cases by year of reporting (n = 97).

Year of Reporting	Number of Cases
2003	2
2004	-
2005	2
2006	2
2007	2
2008	14
2009	2
2010	1
2011	16
2012	3
2013	15
2014	14
2015	3
2016	3
2017	10
2018	1
2019	9
Total	99

**Table 4:** Population, proportion of susceptibles of the serosurvey and cumulative incidence 2009–2016 by district.

District	Mean Population 1996–2016	Proportion Susceptibles	Number of Cases	Mean Annual Incidence (2009–2019 *) Cases per 1,000,000 Inhabitants
Imst	56,073	5.0%	4	6.74
Innsbruck Land	164,010	5.6%	13	7.49
Innsbruck Stadt	116,202	6.0%	17	13.82
Kitzbühel	69,991	**8.6%**	15	**20.25**
Kufstein	99,433	6.9%	19	18.06
Landeck	44,092	6.9%	0	0.00
Lienz	49,783	7.2%	2	3.80
Reutte	31,729	5.4%	5	14.89
Schwaz	78,303	5.8%	2	2.32
Total	709,616		77	9.86

* = as of 20 July 2019.

## Supplementary Materials

The following are available online at https://www.mdpi.com/1999-4915/11/8/765/s1, Sheet S1: seroprevalence data, Sheet S2: Minimal dataset surv s, Sheet S3: MDS MCV coverage data.
